# Biological impact of iberdomide in patients with active systemic lupus erythematosus

**DOI:** 10.1136/annrheumdis-2022-222212

**Published:** 2022-04-27

**Authors:** Peter E Lipsky, Ronald van Vollenhoven, Thomas Dörner, Victoria P Werth, Joan T Merrill, Richard Furie, Milan Petronijevic, Benito Velasco Zamora, Maria Majdan, Fedra Irazoque-Palazuelos, Robert Terbrueggen, Nikolay Delev, Michael Weiswasser, Shimon Korish, Mark Stern, Sarah Hersey, Ying Ye, Allison Gaudy, Zhaohui Liu, Robert Gagnon, Shaojun Tang, Peter H Schafer

**Affiliations:** 1 RILITE Foundation and AMPEL BioSolutions, Charlottesville, Virginia, USA; 2 Amsterdam University Medical Centers, Amsterdam, The Netherlands; 3 German Rheumatism Research Center, Charité University Hospital, Berlin, Germany; 4 University of Pennsylvania and the Michael J. Crescenz VA Medical Center, Philadelphia, Pennsylvania, USA; 5 Oklahoma Medical Research Foundation, Oklahoma City, Oklahoma, USA; 6 Department of Rheumatology, Northwell Health, Great Neck, New York, USA; 7 Military Medical Academy, Belgrade, Serbia; 8 Instituto CER S.A, Buenos Aires, Argentina; 9 Samodzielny Publiczny Szpital Kliniczny Nr 4 w Lublinie, Medical University of Lublin, Lublin, Poland; 10 Centro de Investigación y Tratamiento Reumatológico SC, Mexico City, Mexico; 11 DxTerity Diagnostics, Rancho Dominguez, California, USA; 12 Bristol Myers Squibb, Princeton, New Jersey, USA

**Keywords:** lupus Erythematosus, Systemic, B-Lymphocytes, immune system diseases

## Abstract

**Objectives:**

Iberdomide is a high-affinity cereblon ligand that promotes proteasomal degradation of transcription factors Ikaros (*IKZF1*) and Aiolos (*IKZF3*). Pharmacodynamics and pharmacokinetics of oral iberdomide were evaluated in a phase 2b study of patients with active systemic lupus erythematosus (SLE).

**Methods:**

Adults with autoantibody-positive SLE were randomised to placebo (n=83) or once daily iberdomide 0.15 mg (n=42), 0.3 mg (n=82) or 0.45 mg (n=81). Pharmacodynamic changes in whole blood leucocytes were measured by flow cytometry, regulatory T cells (Tregs) by epigenetic assay, plasma cytokines by ultrasensitive cytokine assay and gene expression by Modular Immune Profiling.

**Results:**

Iberdomide exhibited linear pharmacokinetics and dose-dependently modulated leucocytes and cytokines. Compared with placebo at week 24, iberdomide 0.45 mg significantly (p<0.001) reduced B cells, including those expressing CD268 (TNFRSF13C) (−58.3%), and plasmacytoid dendritic cells (−73.9%), and increased Tregs (+104.9%) and interleukin 2 (IL-2) (+144.1%). Clinical efficacy was previously reported in patients with high *IKZF3* expression and high type I interferon (IFN) signature at baseline and confirmed here in those with an especially high IFN signature. Iberdomide decreased the type I IFN gene signature only in patients with high expression at baseline (−81.5%; p<0.001) but decreased other gene signatures in all patients.

**Conclusion:**

Iberdomide significantly reduced activity of type I IFN and B cell pathways, and increased IL-2 and Tregs, suggesting a selective rebalancing of immune abnormalities in SLE. Clinical efficacy corresponded to reduction of the type I IFN gene signature.

**Trial registration number:**

NCT03161483.

**Key messages**

**What is already known about this subject?**

- Iberdomide is a high-affinity cereblon ligand which promotes proteasomal degradation of Ikaros (*IKZF1*) and Aiolos (*IKZF3*) and is currently in development for the treatment of patients with systemic lupus erythematosus (SLE), multiple myeloma and lymphoma.
- In a phase 2a trial in patients with active SLE, iberdomide significantly reduced B cells and plasmacytoid dendritic cells (pDCs) and showed trends of improvements in SLE disease severity.

**What does this study add?**

- In this larger phase 2b study, iberdomide significantly improved lupus disease activity and reduced hallmarks of the immunopathogenesis of SLE by decreasing B cells, pDCs and myeloid dendritic cells, and by increasing interleukin 2 and regulatory T cells.
- In patients with a high type I interferon (IFN) gene signature at baseline, iberdomide treatment reduced the IFN gene signature score by as much as 81% from the median at baseline, an effect that coincided with an improved SLE Responder Index-4 clinical response rate.

**How might this impact on clinical practice or future developments?**

- This study confirmed the mechanism of action of iberdomide in vivo in patients with SLE and identified the high type I IFN gene signature as a predictive biomarker for evaluation as a selection tool in future clinical studies of iberdomide.

## Introduction

Systemic lupus erythematosus (SLE) is a heterogeneous autoimmune inflammatory disorder arising from the interaction of a genetically determined immune phenotype with environmental factors.^1 2^ Disease susceptibility is influenced by genes related to immune response pathways and major histocompatibility complex classes I and II. Dysregulated immune responses lead to B cell hyperactivity and production of pathogenic autoantibodies. Immune complexes containing nucleic acids are potential stimuli of the innate immune system, leading to type I interferon (IFN) production in SLE.

Ikaros (*IKZF1*) and Aiolos (*IKZF3*) are zinc finger transcription factors involved in immune cell development and homeostasis.^3–5^ Ikaros is required for development of B cells and plasmacytoid dendritic cells (pDCs), which are important producers of IFN-α. Ikaros also represses interleukin 2 (IL-2) transcription.^6^ Aiolos is a B cell modulator and is required for maturation of plasma cells. *IKZF1* and *IKZF3* mRNA and proteins are overexpressed in the cells of patients with SLE.^4 5 7–10^ Genetic variants in the *IKZF1* and *IKZF3* loci are associated with an increased risk of developing SLE.^2 10^ In particular, the *IKZF1* polymorphism rs4917014 was identified as a trans-expression quantitative trait locus (eQTL) increasing expression of type I IFN response genes (*HERC5*, *IFI6*, *IFIT1*, *MX1* and *TNFRSF21*).^9^

Iberdomide (CC-220) is a high-affinity cereblon ligand, which promotes ubiquitination and proteasomal degradation of Ikaros and Aiolos.^4 5 11^ The binding affinity of iberdomide to cereblon is higher than that of other related cereblon binders, such as lenalidomide or pomalidomide. In vitro studies have shown a potent effect of iberdomide in reducing Ikaros and Aiolos protein levels in B cells, T cells and monocytes from healthy donors. In peripheral blood mononuclear cells from patients with SLE, iberdomide inhibited autoantibody production and B cell differentiation. Iberdomide also increased T cell-derived IL-2 production in the whole blood of healthy volunteers owing to an iberdomide-mediated decrease in the repressive activity of Ikaros and Aiolos.^5^ In a pilot phase 2 trial of ascending doses of iberdomide in patients with SLE, strong correlations were observed between iberdomide exposure and reductions in the numbers of B cells and pDCs.^12^

A phase 2 randomised, controlled trial evaluated the efficacy and safety of iberdomide compared with placebo over 24 weeks in patients with active SLE. As reported elsewhere,^13^ the primary efficacy endpoint of SLE Responder Index-4 (SRI-4) response was met with 54% of patients receiving iberdomide 0.45 mg once daily having achieved an SRI-4 response versus 35% in the placebo group (stratified difference: 19.4%; 95% CI 4.1 to 33.4; p=0.01) at week 24. Furthermore, the treatment effect of iberdomide 0.45 mg compared with placebo for SRI-4 response was greater in the prespecified biomarker-defined subsets of patients with high expression of *IKZF3* at baseline (64% vs 33%; p=0.011) and high expression of type I IFN at baseline (60% vs 33%; p=0.006). As understanding of SLE pathophysiology increases, the precise biological impact of therapeutic agents is of great interest and may be useful in identifying biomarkers of clinical response. Therefore, the effects of iberdomide on immunologic biomarkers in patients with active SLE were further evaluated in this phase 2 study.

## Patients and methods

### Study design

The study design of the phase 2, multinational, randomised, placebo-controlled, double-blind study has been reported.^13^ Briefly, patients with active SLE were randomised (2:2:1:2) to receive oral iberdomide (0.45 mg, 0.3 mg or 0.15 mg) or placebo once daily for 24 weeks while continuing standard-of-care medications.

### Patients

Eligible patients were adults (≥18 years of age) with a diagnosis of SLE for at least 6 months, a Systemic Lupus Erythematosus Disease Activity Index 2000 score ≥6 points and positive for autoantibodies associated with SLE. Stable doses of corticosteroids (≤20 mg prednisone or equivalent daily) were allowed. Exclusion criteria were active, severe or unstable neuropsychiatric lupus disease, antiphospholipid syndrome or history of thrombosis, estimated glomerular filtration rate <45 mL/min/1.7 m^2^ or proteinuria >2000 mg/d, or active lupus nephritis, which may require induction therapy.

### Pharmacokinetic assessments

One predose blood sample was collected at week 4, week 12 and week 24 for pharmacokinetic analysis. Iberdomide concentration was determined by a validated assay.^14^ A population pharmacokinetic analysis was performed (see online supplemental methods), and individual oral clearance values were used to calculate area under the concentration–time curve.

10.1136/annrheumdis-2022-222212.supp4 Supplementary data

### Pharmacodynamic assessments

Blood samples were collected at baseline, week 4, week 12 and week 24 for analysis of whole blood leucocytes, plasma proteins and whole blood gene expression. Flow cytometry (Covance, Indianapolis, Indiana, USA) was used to analyse B cells (CD19+ and CD20+), T cells (including CD4+ and CD8+), plasmablasts, pDCs and myeloid dendritic cells (mDCs). T helper 17 (Th17) cells, regulatory T cells (Tregs) and T follicular helper (Tfh) cells were measured by epigenetic assays (Epiontis ID; Epiontis GmbH, Berlin, Germany), an approach that correlates strongly with flow cytometry.^15–17^

Plasma cytokines, IL-2, IL-10, IL-17A, IL-17F and the B lymphocyte stimulator (BLyS; *TNFSF13b*) were determined by the ultrasensitive Singulex assay (Erenna; EMD Millipore, Burlington, Massachusetts, USA). The DxTerity Autoimmune Profiler (DxTerity, Rancho Dominguez, California, USA) was used to analyse whole blood stabilised through direct collection into DxCollect tubes for subsequent gene expression using chemical ligation probe amplification technology for generating PCR products. The resultant PCR amplicons were then separated by capillary electrophoresis on the ABI 3500xL Dx Genetic Analyzer (ThermoFisher Scientific, Waltham, Massachusetts, USA) for the following gene modules: B cell (*CD19*, *BACH2* and *CD22*), type I IFN (*IFI27*, *IFI44*, *IFI44L* and *RSAD2* 18), Ikaros (eQTL) type I IFN (*HERC5*, *IFI6*, *IFIT1*, *MX1* and *TNFRSF21* 9) and T cell exhaustion (*CTLA4*, *IL7R*, *LAG3*, *PDCD1* and *ABCE1* 19). Samples were also tested for *IKZF1* (Ikaros) and *IKZF3* (Aiolos) gene expression levels.

Cut-off values for each gene expression module were determined a priori based on an independent training data set from the peripheral blood samples of 96 patients with SLE who were receiving standard-of-care medications but not biologics (DxTerity). An exploratory analysis was conducted on study data for the type I IFN and Aiolos signatures using a bootstrapping and aggregating of thresholds from trees procedure (see online supplemental methods).^20^

Given that greater clinical treatment effect was observed in patient subsets with elevated expression of type I IFN and Aiolos modules, we analysed biomarkers in these subsets at baseline and as median per cent change from baseline.

### Statistical analyses

Pharmacokinetic analyses were performed for all patients who were randomised and received ≥1 dose of iberdomide with ≥1 quantifiable plasma concentration. Pharmacodynamic analyses included patients with a baseline value and a value at the time point reported. Data were reported as adjusted mean per cent changes from baseline. Treatment comparison of adjusted means was based on multiple imputation in conjunction with a regression model that used M-estimation, had the absolute value or change from baseline at a given time point as the response variable and adjusted for treatment group, baseline value and stratification factors. There was no correction for multiple comparisons.

## Results

### Patients

A total of 288 patients received treatment. As reported elsewhere, baseline patient demographics and disease characteristics were balanced between treatment groups.^13^ The proportions of patients with expression of specific gene modules were generally similar between the treatment groups (online supplemental table 1). High Aiolos gene expression was more common in the iberdomide 0.3 mg and 0.45 mg dose groups, and type I IFN module high expression was more common in the 0.45 mg group.

### Pharmacokinetics

Iberdomide exhibited linear pharmacokinetics (online supplemental figure 1A). Exposure increased in a dose-related manner over the dose range of 0.15–0.45 mg once daily, with a 3-fold dose increase resulting in an approximately 2.5-fold increase in the area under the concentration–time curve at steady state. Age, body weight, creatinine clearance, race, sex, ethnicity and disease status did not have a clinically significant effect on iberdomide exposure. There were no differences in iberdomide pharmacokinetics between patients with low and high type I IFN signature or Aiolos expression at baseline (online supplemental figure 1BC).

10.1136/annrheumdis-2022-222212.supp1 Supplementary data

### Pharmacodynamics

**Figure 1:**
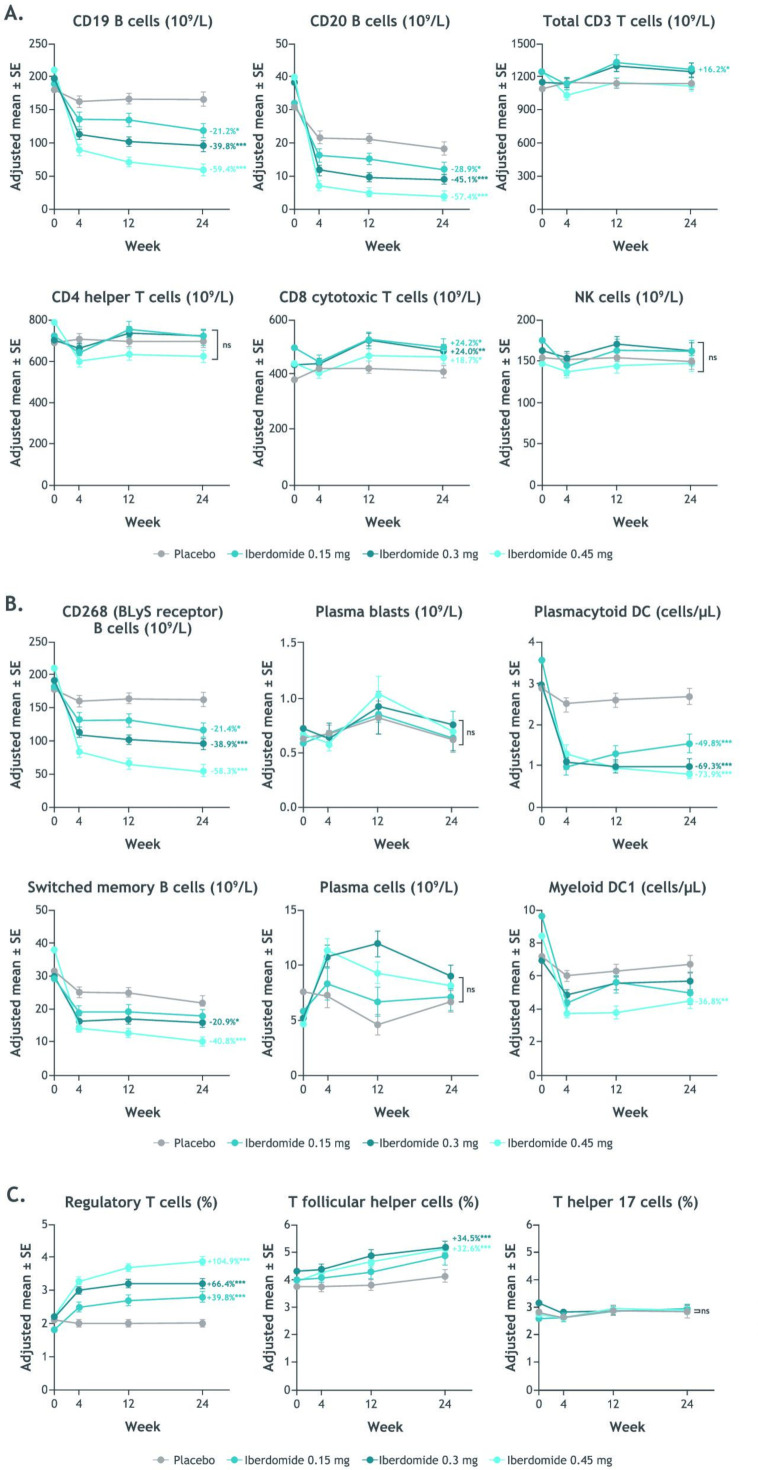
Time course of change from baseline during iberdomide treatment in whole blood leucocyte counts and selected B cells, T cells and NK cells by flow cytometry (Covance, Indianapolis, Indiana, USA) (A), CD268, plasma blasts, switched memory B cells DC subset counts and plasma cells by flow cytometry (B) and Tregs, Tfh cells and Th17 cells by epigenetic assay (Epiontis ID, Epiontis GmbH, Berlin, Germany) (C). *p≤0.05; **p≤0.01; ***p≤0.001 vs placebo. Values shown are the treatment comparison vs placebo of adjusted mean per cent change from baseline. See online supplemental table 2 for numeric data. BLyS, B lymphocyte stimulator; DC, dendritic cell; NK, natural killer; Tfh, T follicular helper; Th17, T helper 17; Tregs, regulatory T cells.

At week 24, iberdomide significantly decreased CD19+ and CD20+ B cells and increased CD8+ cytotoxic T cells from baseline in a dose-dependent manner compared with placebo (figure 1). Iberdomide had no effect on the numbers of CD4+ Th cells or natural killer cells. The difference in adjusted mean per cent change from baseline to week 24 in B cells expressing CD268 (*TNFRSF13C*, encoding BLyS receptor) for iberdomide 0.45 mg compared with placebo was −58.3% (p<0.001) and for post-switched memory B cells was −40.8% (p<0.001). Significant treatment differences for iberdomide 0.45 mg were also noted for pDCs (−73.9%; p<0.001) and mDC 1 cells (−36.8%; p=0.004), Tregs (104.9%; p<0.001) and Tfh cells (+32.6%; p<0.001) at week 24 (figure 1). No significant changes were noted for plasmablasts or plasma cells, which were not significantly elevated at baseline, or Th17 cells.

**Figure 2:**
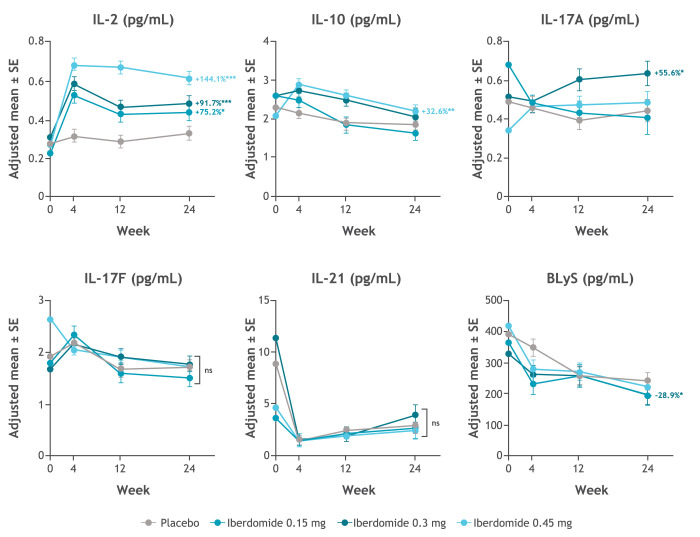
Change from baseline in plasma cytokines during iberdomide treatment by ultrasensitive cytokine assays (Erenna, EMD Millipore, Burlington, Massachusetts, USA). *p≤0.05; **p≤0.01; ***p≤0.001 vs placebo. Values shown are the treatment comparison vs placebo of adjusted mean per cent change from baseline. See online supplemental table 3 for numeric data. BLyS, B lymphocyte stimulator; IL, interleukin.

Iberdomide increased IL-2 levels from baseline compared with placebo (figure 2). Iberdomide treatment resulted in a dose-dependent increase in IL-2, reaching +144.1% for the 0.45 mg dose (p<0.001), +91.7% for the 0.3 mg dose and +75.2% for the 0.15 mg dose versus placebo. No dose-dependent changes in IL-10, IL-17A, IL-17F, IL-21 or BLyS were noted.

**Figure 3:**
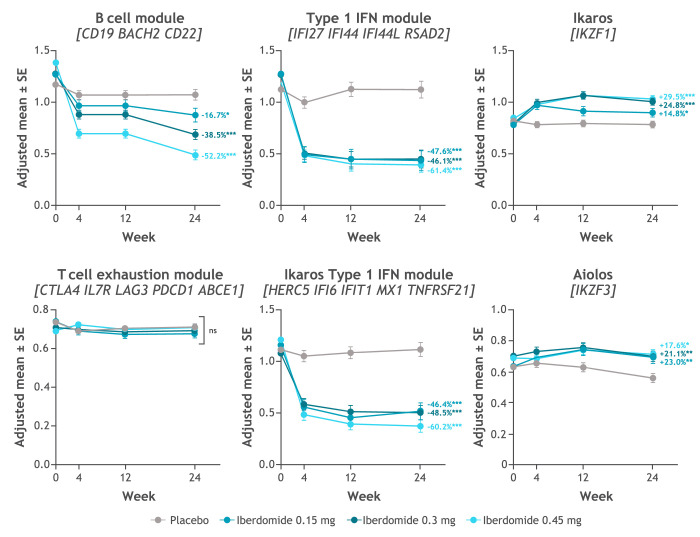
Change from baseline in whole blood gene expression during iberdomide treatment by multiplex PCR-based chemical ligation probe amplification target capture on the ThermoFisher ABI 3500xL DX Genetic Analyzer (DxTerity CLIA-certified laboratory)^a^. *p≤0.05; **p≤0.01; ***p≤0.001. ^a^B cell module: *CD19*, *BACH2* and *CD22*; type I IFN module: *IFI27*, *IFI44*, *IFI44L* and *RSAD2* 18; Ikaros type I IFN module: *HERC5*, *IFI6*, *IFIT1*, *MX1* and *TNFRSF21* 9; and T cell exhaustion module: *CTLA4*, *IL7R*, *LAG3*, *PDCD1* and *ABCE1*.^19^ Values shown are the treatment comparison vs placebo of adjusted mean per cent change from baseline. See online supplemental table 4 for numeric data. IFN, interferon.

Iberdomide decreased expression of gene modules representing the type I IFN, Ikaros eQTL type I IFN gene signature and B cell pathways and increased expression of Ikaros and Aiolos genes (figure 3). A dose–response relationship was noted for the B cell gene module but not for the type I IFN module.

**Figure 4:**
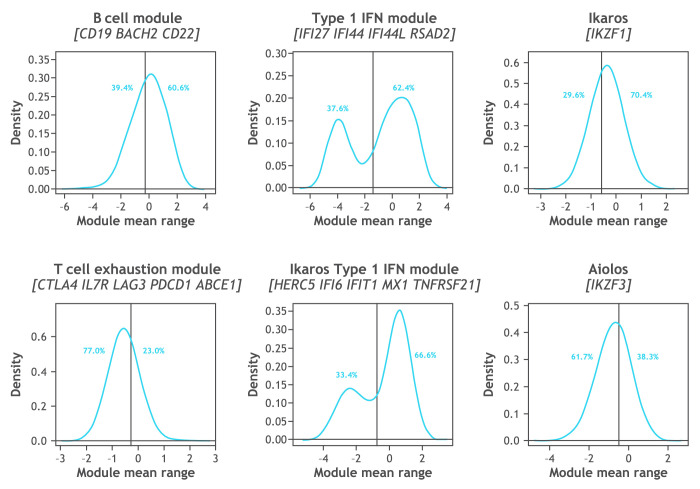
Patient subsets based on peripheral blood gene expression at baseline. The cut-offs were set a priori based on an independent training data set (96 samples from patients with SLE, data not shown). The type I IFN module and the Ikaros type I IFN (eQTL) module had bimodal distributions and the cut-offs were set at the antimode: type I IFN module (*IFI27*, *IFI44*, *IFI44L* and *RSAD2*)=−1.38; Ikaros type I IFN module (*HERC5*, *IFI6*, *IFIT1*, *MX1* and *TNFRSF21*)=−0.76. The distributions of Ikaros, Aiolos and B cell module were unimodal, and the cut-offs were set at the median: Ikaros (*IKZF1*)=−0.58; Aiolos (*IKZF3*)=−0.49; B cell module (*CD19*, *BACH2* and *CD22*)=−0.3; T cell exhaustion module (*CTLA4*, *IL7R*, *LAG3*, *PDCD1* and *ABCE1*)=−0.51. eQTL, expression quantitative trait locus; IFN, interferon.

**Figure 5:**
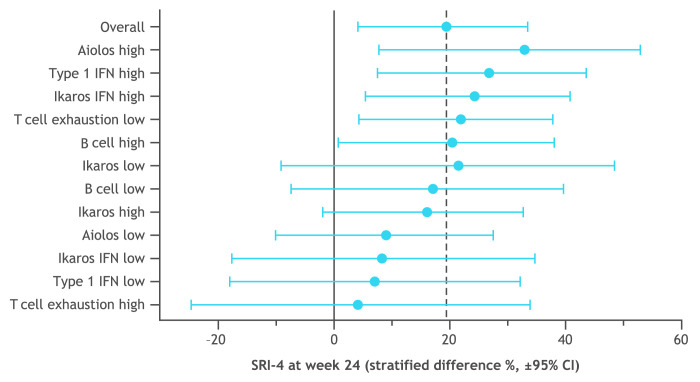
Clinical efficacy treatment comparison (week 24 SRI-4 response rate, iberdomide 0.45 mg—placebo) within prespecified patient subsets defined by gene expression at baseline. Gene module score cut-offs were set as described in figure 5. See online supplemental table 5 for numeric data. IFN, interferon; SLE, systemic lupus erythematosus; SRI-4, SLE Responder Index-4.

**Figure 6:**
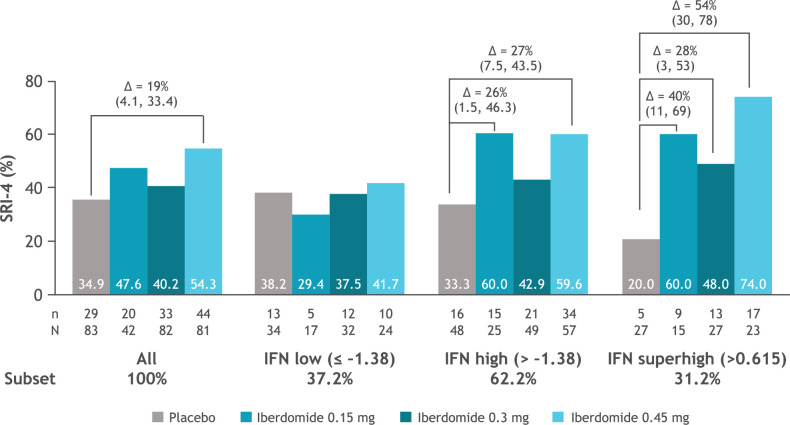
SRI-4 response rate at week 24 in the patient subsets defined by type I IFN gene signature at baseline. Δ=stratified difference from placebo (95% CI); n=number of responders; N=number of patients per subset within each treatment group. IFN, interferon; SLE, systemic lupus erythematosus; SRI-4, SLE Responder Index-4.

**Figure 7:**
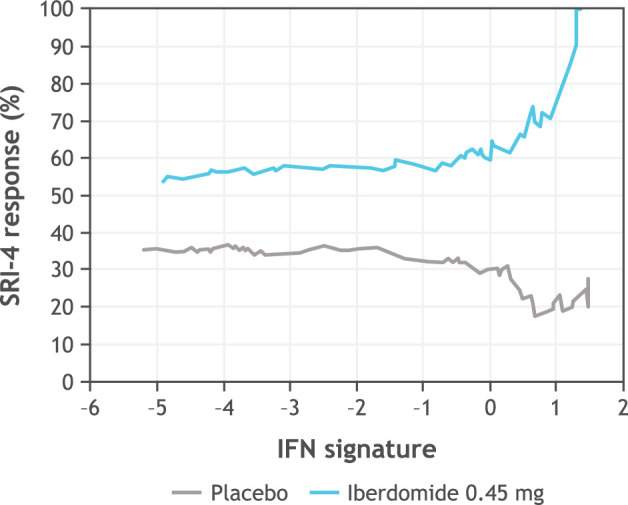
Relationship between baseline type I IFN signature and SRI-4 response rates at week 24 comparing placebo and iberdomide 0.45 mg treated SRI-4 cumulative response rates across the range of baseline type I IFN signature values (*IFI27*, *IFI44*, *IFI44L* and *RSAD2*). In exploratory analysis using bootstrapping and aggregating of thresholds from trees, the type I IFN signature optimal cut point was at 0.615 (interaction p=0.0037), SRI-4 at 0.45 mg=74% vs placebo=20%, OR=11.3 (2.9–43.8). this ‘IFN-Superhigh’ cut point captured 90/288 (31%) patients. At the extreme IFN >1.31 (top 14% of patients), in the iberdomide 0.45 mg group, 11/11 (100%) patients had an SRI-4 response. IFN, interferon; SLE, systemic lupus erythematosus; SRI-4, SLE Responder Index-4.

The distribution of patient subsets by gene expression at baseline is shown in figure 4 with the type I IFN signature showing a biphasic distribution. Greater SRI-4 responses were noted in subsets having a high level of Aiolos and type I IFN gene expression at baseline (figure 5). In an exploratory analysis, the subset of patients in the 0.45 mg group with the highest expression of the type I IFN signature (baseline type I IFN gene signature >0.615) was found to have an SRI-4 response rate treatment difference of 54% at week 24 versus placebo (figure 6). Response rate plots (figure 7) showed that as the baseline IFN gene signature increased in magnitude, the week 24 SRI-4 response increased for iberdomide 0.45 mg up to 100% but decreased for placebo.

Analysis of changes in pharmacodynamic markers showed that patients with a high baseline type I IFN signature manifested a significant reduction in the IFN signature as a result of active treatment, whereas those with low baseline IFN signature did not. In contrast, both groups exhibited a significant reduction in B cells and pDCs and significant increases in IL-2 and Tregs (online supplemental figure 2). Baseline Aiolos expression had no impact on changes in type I IFN signature or any other pharmacodynamic parameter (online supplemental figure 3).

10.1136/annrheumdis-2022-222212.supp2 Supplementary data

10.1136/annrheumdis-2022-222212.supp3 Supplementary data

## Discussion

Pharmacodynamic analyses showed that iberdomide treatment reduced activity of the B cell and type I IFN pathways. These effects were evident in reductions in total B cells and B cells expressing the gene for the BLyS receptor and in switched memory B cells. Elevated BLyS levels have been documented in patients with SLE and shown to correlate with disease activity.^21^ BLyS also induces plasmablast differentiation and drives autoantibody production in SLE.^4 21^ Among patients with elevated anti-dsDNA antibodies at baseline, higher doses of iberdomide (0.3 mg and 0.45 mg) resulted in dose-dependent reductions versus placebo at week 24. Although there was no change in plasma cells in the blood, there may have been a change in plasma cell production of autoantibodies and/or plasma cells located in tissues. Treatment with iberdomide was associated with a significant, dose-dependent reduction in pDCs and mDCs, which are primary sources of type I IFNs.^1^

In patients with SLE, levels of IL-2 have been reported to vary.^4 22^ A reduction in IL-2 production from T cells has been associated with impaired Treg development. Iberdomide has been shown to increase IL-2 production from T cells^5^ and, in this clinical trial of SLE patients, iberdomide increased serum levels of IL-2 and expanded the Treg population in the blood. Ikaros is a repressor of IL-2 gene transcription,^6 23^ and, therefore, reduction of Ikaros protein would be expected to result in transcriptional de-repression and an increase in IL-2 production. Because IL-2 is a major driver of Treg expansion and maintenance,^24^ the observed increase in Tregs (up to +104.9%) could be explained by the increase in IL-2 (+144.1%). Besides the increase in IL-2, there were no dose-dependent effects of iberdomide on the other cytokines measured (IL-10, IL-17A, IL-17F, IL-21 and BLyS). No effect of iberdomide was observed on IL-17 plasma levels or Th17 cells, consistent with a lack of effect on the Th17 immune response in patients with lupus. These effects confirm the unique mechanism of action of iberdomide, suppressing dendritic cells and the type I IFN response, reducing B cells and anti-dsDNA antibodies, and augmenting IL-2 and Tregs, consistent with the role of Ikaros and Aiolos in immune homeostasis and with prior studies in healthy volunteers and patients with SLE.^4 5^ The increase in *IKZF1* and *IKZF3* gene expression by iberdomide may be explained by the negative feedback each transcription factor can have on its own expression.^25^

The majority of patients enrolled in this trial had elevated expression of genes in the type I IFN and Ikaros pathways, which are typical of the SLE population.^26^ Dysregulation of the type I IFN pathway can contribute to clinical features, immune dysregulation and laboratory manifestations in SLE.^27^ However, the strongest association to gene expression changes is found with autoantibodies, which are influenced by patient ancestry.^28^ In addition, patients with active SLE have decreased Treg numbers and function, as excess IFN prevents normal activation and expansion of Tregs in response to inflammation.^26^ In the current study, a correlation analysis of baseline variations in gene expression with clinical features found that the type I IFN gene module was directly proportional to SLEDAI and Cutaneous Lupus Erythematosus Disease Area and Severity Index (CLASI) score, and was higher in patients on oral corticosteroids or azathioprine. This is consistent with previous literature associating the type I IFN gene signature with more severe disease and use of corticosteroids and immunosuppressants.^29^ Baseline Aiolos (*IKZF3*) gene expression was not proportional to SLEDAI or CLASI score and was not different in any subgroups based on medication (data not shown).

As previously reported,^13^ iberdomide decreased anti-dsDNA antibodies among patients with high levels at baseline (≥8 IU/mL), with 0.45 mg decreasing levels by 61.2% (p=0.008) and 0.3 mg decreasing levels by 56.1% (p=0.027) compared with placebo. The clinical efficacy of iberdomide in patients with active SLE in this phase 2 study was greater among subgroups who had high expression of the type I IFN or Aiolos gene signature at baseline.^13^ Moreover, exploratory analysis indicates that the highest cut point for the type I IFN subgroup (representing 31% of the total study population) was associated with the most enhanced relationship with response, providing a treatment difference of 54% versus placebo. At the extreme high IFN gene signature (expressed by 14% of patients), 100% (11/11) of patients had an SRI-4 response to iberdomide 0.45 mg, suggesting that the SRI-4 clinical response rate to iberdomide is proportionate to the baseline expression level of the type I IFN gene signature. Iberdomide significantly decreased the type I IFN gene signature only in the IFN-high patient subgroup, which corresponded to stratified treatment differences for SRI-4 in the IFN-high group ranging from 25.6% to 26.8% versus placebo. Iberdomide did not significantly reduce the type I IFN gene signature in the IFN-low patient population, with no significant differences in SRI-4 from placebo in this subgroup. In other studies, the relationship between IFN gene signature and disease activity has varied, a finding that is likely a result of disease and gene expression heterogeneity as well as differences in the methods used to define gene signatures across studies. In several cross-sectional gene expression studies, the type I IFN gene signature has identified a distinct subset of lupus patients who have greater disease severity and a worse clinical prognosis.^30 31^ In a recent longitudinal study, the type I IFN gene signature was prognostic for early development of lupus nephritis after adjusting for age at SLE diagnosis, gender and race (HR: 3.36).^32^

The pharmacodynamic and pharmacokinetic analyses were conducted based on 24 weeks of iberdomide treatment. Longer-term treatment or discontinuation effects were not evaluated. Patients continued to receive standard-of-care medications, including corticosteroids with no mandatory tapering, but the results of iberdomide pharmacodynamic analyses were as predicted, suggesting that background treatment did not impact results. Other factors, including concomitant medication use (antimalarials and immunosuppressants) and ancestral diversity, may impact our findings. Additional analyses of the pharmacodynamic effects could examine the influence of baseline disease characteristics and other response measurements. Results of exploratory cut point analyses require validation in future studies.

The most common adverse events with iberdomide (urinary tract infection, upper respiratory tract infection, neutropenia, influenza, nasopharyngitis and diarrhoea)^13^ might be related to the modulatory effects of iberdomide on innate or adaptive immunity.

In conclusion, iberdomide showed significant improvement in the treatment of patients with active SLE.^12 13^ Predominant pharmacologic activity was observed on the type I IFN and B cell/plasma cell pathways, leading to reductions in B cells, pDCs and autoantibody levels. Increased levels of Tregs and IL-2 suggest immune system rebalancing. An elevated type I IFN gene signature was associated with improved response and the largest change from baseline to week 24 in the gene signature expression. These findings may provide an opportunity to implement precision medicine to evaluate therapy on a molecular basis and potentially identify biomarkers associated with response to iberdomide for evaluation in future clinical studies.

## Data Availability

The Bristol Myers Squibb policy on data sharing may be found at https://www.bms.com/researchers-and-partners/independent-research/data-sharing-request-process.html
