# Supplementary material for: Biological impact of iberdomide in patients with active systemic lupus erythematosus
Source: Ann Rheum Dis. 2022 Apr 27;81(8):1136–42. doi: 10.1136/annrheumdis-2022-222212 (PMC9279852; doi:10.1136/annrheumdis-2022-222212)
Supplement: Supplementary data [file annrheumdis-2022-222212supp004.pdf]

BIOLOGICAL IMPACT OF IBERDOMIDE IN PATIENTS WITH ACTIVE SYSTEMIC LUPUS ERYTHEMATOSUS

Peter E. Lipsky,[1] Ronald van Vollenhoven,[2] Thomas Dörner,[3] Victoria P. Werth,[4] Joan T. Merrill,[5] Richard Furie,[6] Milan Petronijevic,[7] Benito Velasco Zamora,[8] Maria Majdan,[9] Fedra Irazoque-Palazuelos,[10] Robert Terbrueggen,[11] Nikolay Delev,[12] Michael Weiswasser,[12] Shimon Korish,[12] Mark Stern,[12] Sarah Hersey,[12] Ying Ye,[12] Allison Gaudy,[12] Zhaohui Liu,[12] Robert Gagnon,[12] Shaojun Tang,[12*] Peter H. Schafer[12]

[1] RILITE Foundation and AMPEL BioSolutions, Charlottesville, VA, USA; [2]Amsterdam University Medical Centers, Amsterdam, The Netherlands; [3]German Rheumatism Research Center and Charité University Hospitals, Berlin, Germany; [4]University of Pennsylvania and the Michael J. Crescenz VA Medical Center, Philadelphia, PA, USA; [5]Oklahoma Medical Research Foundation, Oklahoma City, OK, USA; [6]Northwell Health, Great Neck, NY, USA; [7]Military Medical Academy, Belgrade, Serbia; [8]Instituto CER S.A., Buenos Aires, Argentina; [9]Samodzielny Publiczny Szpital Kliniczny Nr 4 w Lublinie, Medical University of Lublin, Lublin, Poland; [10]Centro de Investigación y Tratamiento Reumatológico S.C, Miguel Hidalgo, Mexico; [11]DxTerity Diagnostics Inc, Rancho Dominguez, CA, USA; [12]Bristol Myers Squibb, Princeton, NJ, USA

*At the time of the study.

Lipsky PE, *et al*. *Ann Rheum Dis* 2022; 81:1136–1142. doi: 10.1136/annrheumdis-2022-222212

Supplementary Methods

Cut point determination

In this training dataset, the type I IFN module and the Ikaros type I IFN module (genes acting with IKZF1 as expression quantitative trait loci [eQTL]) had bimodal distributions, and the cut points were set at the antimode (i.e., the lowest point in the distribution curve between the two modes): type I IFN module −1.38 and Ikaros type I IFN module −0.76. The distributions of *IKZF1*, *IKZF3*, the B cell module and T cell exhaustion module were unimodal, and the cut points were set at the median (*IKZF1*, −0.58; *IKZF3*, −0.49; B cell module, −0.3; T cell exhaustion module, −0.51).

Exploratory analyses

The bootstrapping and aggregating of thresholds from trees (BATTing) procedure is designed to provide a robust cut point estimate for the single marker being evaluated (e.g., type I IFN). The original data (placebo + 0.45 mg dose groups, with total N from these groups noted as N_{total}) is bootstrap sampled (randomly sampled with size N_{total}, with replacement), and the optimal cut point (C) forms the "tree" by partitioning the data into groups above and below C. C is equivalent to the minimum *P* value for interaction of placebo and iberdomide 0.45 mg SRI-4 response rates in a logistic regression setting, for groups above and below C in the bootstrapped sample. The bootstrapping step is repeated B times (B=1000), and the B values of C are aggregated, with the median of these values determining the optimal cut point. Due to bootstrapping, the threshold is robust to small perturbations, or outliers, in the data and not prone to overfitting. The other dose groups (0.15 and 0.30 mg) are then tested using the optimal

Lipsky PE, *et al. Ann Rheum Dis* 2022; 81:1136–1142. doi: 10.1136/annrheumdis-2022-222212

cut point, for validation of the result. This analysis identified a higher type I IFN cutoff point of 0.615, hereafter referred to as IFN super-high.

Pharmacokinetics

Sparse pharmacokinetic data were pooled with pharmacokinetic data collected previously from healthy subjects and patients with SLE and analysed using population methods to determine individual pharmacokinetic exposure parameters and explore the effect of covariates on exposure.

Supplementary Table 1. Baseline mRNA and DNA expression by treatment group (ITT population)

		Iberdomide		
	**Placebo**	**0.15 mg QD**	**0.3 mg QD**	**0.45 mg QD**
**Patients, n (%)**	**(n=83)**	**(n=42)**	**(n=82)**	**(n=81)**
Ikaros (*IKZF1*) high	56 (68)	28 (67)	53 (65)	64 (79)
Aiolos (*IKZF3*) high	27 (33)	14 (33)	32 (39)	36 (44)
Type I IFN module high	48 (58)	25 (60)	49 (60)	57 (70)
Ikaros IFN module high	49 (59)	30 (71)	49 (60)	62 (77)
T cell exhaustion module low	60 (72)	29 (69)	65 (79)	66 (82)
B cell module high	46 (55)	24 (57)	48 (59)	54 (67)

The cutoffs were set a priori based on an independent training data set. The type I IFN module and the Ikaros type I IFN (eQTL) module had bimodal distributions and the cutoffs were set at the antimode: type I IFN module [*IFI27, IFI44, IFI44L, RSAD2*]=, −1.38, and Ikaros type I IFN module [*HERC5, IFI6, IFIT1, MX1, TNFRSF21*]=−0.76. The distributions of Ikaros, Aiolos and B cell modules were unimodal, and the cutoffs were set at the median: Ikaros [*IKZF1*]=−0.58; Aiolos [*IKZF3*]=−0.49; B cell module [*CD19, BACH2, CD22*]=−0.3; and T cell exhaustion [*CTLA4, IL7R, LAG3, PDCD1, ABCE1*] =−0.51.

IFN, interferon; ITT, intent-to-treat; QD, once daily.

Supplementary Table 2. Numeric data for Figure 1: Time course of change from baseline during iberdomide treatment in selected blood cells

Adjusted mean (SE)	Week 0	Week 4	Week 12	Week 24
CD19 B cells (10^9/L)				
Placebo	180.103	163.211 (8.715)	166.824 (7.430)	166.903 (10.817)
Iberdomide 0.15 mg QD	188.639	136.145 (10.893)	134.922 (10.042)	119.444 (11.039)
Iberdomide 0.3 mg QD	197.616	113.232 (8.174)	102.133 (7.581)	96.007 (8.574)
Iberdomide 0.45 mg QD	211.972	90.019 (8.488)	71.137 (7.697)	60.083 (9.124)
CD20 B cells (10^9/L)				
Placebo	30.492	21.640 (1.826)	21.060 (1.488)	18.210 (1.926)
Iberdomide 0.15 mg QD	31.674	16.127 (2.101)	15.098 (1.759)	12.212 (1.819)
Iberdomide 0.3 mg QD	38.912	11.782 (1.553)	9.653 (1.267)	8.940 (1.381)
Iberdomide 0.45 mg QD	39.914	7.217 (1.496)	5.056 (1.303)	4.038 (1.430)
Total CD3 T cells (10^9/L)				
Placebo	1086.059	1145.725 (38.715)	1134.861 (44.756)	1133.939 (51.177)
Iberdomide 0.15 mg QD	1237.639	1128.985 (54.688)	1324.183 (66.503)	1256.970 (63.590)
Iberdomide 0.3 mg QD	1151.014	1134.530 (38.814)	1291.049 (47.814)	1236.539 (49.437)
Iberdomide 0.45 mg QD	1250.183	1031.406 (40.670)	1148.483 (47.101)	1112.613 (47.559)

Lipsky PE, *et al. Ann Rheum Dis* 2022; 81:1136–1142. doi: 10.1136/annrheumdis-2022-222212

CD4 helper T cells (10^9/L)				
Placebo	681.588	701.100 (23.746)	691.668 (26.724)	692.040 (31.989)
Iberdomide 0.15 mg QD	719.222	637.010 (33.878)	747.584 (39.111)	712.739 (38.355)
Iberdomide 0.3 mg QD	695.904	656.170 (23.997)	732.973 (28.278)	715.382 (29.039)
Iberdomide 0.45 mg QD	787.465	598.295 (24.476)	630.336 (28.022)	618.636 (28.474)
CD8 cytotoxic T cells (10^9/L)				
Placebo	380.912	422.735 (17.031)	424.266 (20.412)	413.080 (22.862)
Iberdomide 0.15 mg QD	498.028	445.980 (24.328)	524.817 (30.733)	498.460 (29.554)
Iberdomide 0.3 mg QD	435.137	439.529 (17.341)	527.051 (22.918)	484.170 (23.982)
Iberdomide 0.45 mg QD	445.352	405.957 (17.421)	472.78 (22.190)	463.766 (24.543)
NK cells (10^9/L)				
Placebo	154.912	152.525 (7.752)	154.705 (8.849)	150.036 (9.796)
Iberdomide 0.15 mg QD	175.722	144.369 (10.529)	163.395 (12.442)	162.187 (13.197)
Iberdomide 0.3 mg QD	163.096	154.067 (7.696)	171.117 (9.682)	161.796 (10.747)
Iberdomide 0.45 mg QD	148.169	138.913 (7.571)	145.904 (8.928)	147.980 (9.531)

Lipsky PE, *et al. Ann Rheum Dis* 2022; 81:1136–1142. doi: 10.1136/annrheumdis-2022-222212

CD268 B cells (10^9/L)				
Placebo	178.196	161.419 (9.091)	163.784 (7.711)	163.015 (10.194)
Iberdomide 0.15 mg QD	181.091	133.339 (11.168)	132.239 (9.999)	117.577 (10.836)
Iberdomide 0.3 mg QD	191.328	112.763 (8.266)	103.979 (7.698)	96.794 (8.432)
Iberdomide 0.45 mg QD	212.301	86.103 (8.919)	67.928 (7.891)	57.405 (9.135)
Plasma blasts (10^9/L)				
Placebo	0.645	0.681 (0.075)	0.827 (0.154)	0.629 (0.123)
Iberdomide 0.15 mg QD	0.601	0.682 (0.093)	0.853 (0.179)	0.636 (0.116)
Iberdomide 0.3 mg QD	0.722	0.640 (0.069)	0.926 (0.143)	0.758 (0.127)
Iberdomide 0.45 mg QD	0.664	0.595 (0.073)	1.043 (0.163)	0.707 (0.097)
Plasmacytoid DC (cells/μL)				
Placebo	2.975	2.525 (0.165)	2.567 (0.162)	2.719 (0.206)
Iberdomide 0.15 mg QD	3.614	1.045 (0.214)	1.315 (0.206)	1.555 (0.229)
Iberdomide 0.3 mg QD	3.007	1.116 (0.175)	1.030 (0.157)	1.012 (0.173)
Iberdomide 0.45 mg QD	3.000	1.323 (0.189)	1.011 (0.165)	0.866 (0.169)

Lipsky PE, *et al. Ann Rheum Dis* 2022; 81:1136–1142. doi: 10.1136/annrheumdis-2022-222212

Switched memory B cells (10^9/L)				
Placebo	31.319	25.191 (1.486)	25.173 (1.375)	22.080 (2.001)
Iberdomide 0.15 mg QD	29.476	19.110 (1.973)	19.427 (1.995)	18.126 (1.805)
Iberdomide 0.3 mg QD	31.954	16.833 (1.451)	17.131 (1.361)	16.150 (1.411)
Iberdomide 0.45 mg QD	38.573	14.698 (1.455)	12.902 (1.412)	10.474 (1.396)
Plasma cells (10^9/L)				
Placebo	7.606	7.243 (1.075)	4.603 (0.919)	6.744 (1.013)
Iberdomide 0.15 mg QD	5.801	8.282 (1.397)	6.650 (1.300)	7.022 (1.135)
Iberdomide 0.3 mg QD	5.057	10.824 (0.994)	11.939 (1.173)	8.941 (1.064)
Iberdomide 0.45 mg QD	4.789	11.394 (1.014)	9.309 (0.979)	8.413 (0.957)
Myeloid DC1 (cells/μL)				
Placebo	7.615	6.025 (0.365)	6.360 (0.437)	6.736 (0.523)
Iberdomide 0.15 mg QD	9.650	4.330 (0.502)	5.630 (0.589)	5.020 (0.701)
Iberdomide 0.3 mg QD	7.071	4.818 (0.342)	5.605 (0.451)	5.721 (0.527)
Iberdomide 0.45 mg QD	8.549	3.837 (0.346)	3.830 (0.392)	4.532 (0.460)

Lipsky PE, *et al. Ann Rheum Dis* 2022; 81:1136–1142. doi: 10.1136/annrheumdis-2022-222212

Regulatory T cells (%)				
Placebo	2.125	2.035 (0.099)	2.011 (0.113)	1.996 (0.125)
Iberdomide 0.15 mg QD	1.845	2.484 (0.138)	2.672 (0.159)	2.762 (0.168)
Iberdomide 0.3 mg QD	2.240	3.039 (0.110)	3.231 (0.132)	3.255 (0.153)
Iberdomide 0.45 mg QD	2.173	3.293 (0.102)	3.674 (0.117)	3.900 (0.132)
T follicular helper cells (%)				
Placebo	3.818	3.760 (0.162)	3.788 (0.184)	4.150 (0.229)
Iberdomide 0.15 mg QD	4.049	4.145 (0.232)	4.284 (0.254)	4.863 (0.304)
Iberdomide 0.3 mg QD	4.276	4.368 (0.164)	4.911 (0.197)	5.200 (0.234)
Iberdomide 0.45 mg QD	3.896	4.305 (0.161)	4.683 (0.187)	5.189 (0.227)
T helper 17 cells (%)				
Placebo	2.764	2.613 (0.108)	2.865 (0.116)	2.837 (0.137)
Iberdomide 0.15 mg QD	2.615	2.623 (0.157)	2.878 (0.164)	2.965 (0.181)
Iberdomide 0.3 mg QD	3.141	2.781 (0.111)	2.938 (0.126)	2.874 (0.143)
Iberdomide 0.45 mg QD	2.725	2.596 (0.107)	2.819 (0.118)	2.962 (0.132)

BLyS, B lymphocyte stimulator; DC, dendritic cell; NK, natural killer; SE, standard error.

Lipsky PE, *et al. Ann Rheum Dis* 2022; 81:1136–1142. doi: 10.1136/annrheumdis-2022-222212

Supplementary Table 3. Numeric data for Figure 2: Change from baseline in plasma cytokines during iberdomide treatment by ultra-sensitive cytokine assays

Adjusted mean (SE)	Week 0	Week 4	Week 12	Week 24
IL-2 (pg/mL)				
Placebo	0.277	0.322 (0.033)	0.289 (0.031)	0.333 (0.036)
Iberdomide 0.15 mg QD	0.229	0.535 (0.045)	0.434 (0.043)	0.446 (0.048)
Iberdomide 0.3 mg QD	0.311	0.591 (0.036)	0.468 (0.036)	0.489 (0.041)
Iberdomide 0.45 mg QD	0.282	0.694 (0.033)	0.676 (0.033)	0.622 (0.035)
IL-10 (pg/mL)				
Placebo	2.276	2.151 (0.139)	1.871 (0.150)	1.851 (0.140)
Iberdomide 0.15 mg QD	2.586	2.483 (0.188)	1.828 (0.212)	1.630 (0.183)
Iberdomide 0.3 mg QD	2.593	2.726 (0.146)	2.487 (0.165)	2.031 (0.147)
Iberdomide 0.45 mg QD	2.092	2.889 (0.137)	2.597 (0.146)	2.200 (0.143)
IL-17A (pg/mL)				
Placebo	0.490	0.463 (0.034)	0.392 (0.046)	0.445 (0.061)
Iberdomide 0.15 mg QD	0.683	0.479 (0.044)	0.434 (0.062)	0.408 (0.085)
Iberdomide 0.3 mg QD	0.511	0.492 (0.035)	0.606 (0.054)	0.638 (0.063)
Iberdomide 0.45 mg QD	0.346	0.466 (0.032)	0.475 (0.046)	0.490 (0.057)

IL-17F (pg/mL)				
Placebo	1.921	2.214 (0.110)	1.684 (0.146)	1.715 (0.150)
Iberdomide 0.15 mg QD	1.800	2.347 (0.160)	1.600 (0.193)	1.513 (0.178)
Iberdomide 0.3 mg QD	1.680	2.162 (0.112)	1.915 (0.166)	1.773 (0.158)
Iberdomide 0.45 mg QD	2.650	2.065 (0.112)	1.917 (0.141)	1.736 (0.154)
IL-21 (pg/mL)				
Placebo	8.930	1.653 (0.519)	2.418 (0.418)	3.018 (0.808)
Iberdomide 0.15 mg QD	3.649	1.545 (0.586)	2.207 (0.574)	2.691 (1.077)
Iberdomide 0.3 mg QD	11.487	1.496 (0.538)	1.954 (0.465)	3.994 (1.099)
Iberdomide 0.45 mg QD	4.793	1.417 (0.553)	2.206 (0.420)	2.456 (0.779)
BLyS (pg/mL)				
Placebo	391.910	349.972 (27.884)	257.891 (28.241)	240.733 (28.804)
Iberdomide 0.15 mg QD	366.604	233.758 (36.399)	258.327 (36.882)	194.094 (31.097)
Iberdomide 0.3 mg QD	330.969	262.041 (27.318)	258.493 (30.421)	192.539 (24.621)
Iberdomide 0.45 mg QD	423.164	282.603 (26.910)	271.577 (27.586)	220.855 (24.755)

BLyS, B lymphocyte stimulator; IL, interleukin; SE, standard error.

Lipsky PE, *et al*. *Ann Rheum Dis* 2022; 81:1136–1142. doi: 10.1136/annrheumdis-2022-222212

Supplementary Table 4. Numeric data for Figure 3: Change from baseline in whole blood gene expression during iberdomide treatment

**Adjusted mean (SE)**	**Week 0**	**Week 4**	**Week 12**	**Week 24**
B cell module				
Placebo	1.183	1.077 (0.042)	1.087 (0.051)	1.079 (0.052)
Iberdomide 0.15 mg QD	1.274	0.973 (0.058)	0.974 (0.064)	0.882 (0.066)
Iberdomide 0.3 mg QD	1.280	0.887 (0.043)	0.770 (0.050)	0.693 (0.049)
Iberdomide 0.45 mg QD	1.389	0.702 (0.042)	0.581 (0.048)	0.496 (0.048)
Type 1 IFN module				
Placebo	1.124	0.997 (0.055)	1.126 (0.069)	1.123 (0.081)
Iberdomide 0.15 mg QD	1.273	0.492 (0.078)	0.448 (0.095)	0.434 (0.095)
Iberdomide 0.3 mg QD	1.258	0.507 (0.060)	0.447 (0.075)	0.449 (0.087)
Iberdomide 0.45 mg QD	1.191	0.482 (0.055)	0.402 (0.070)	0.392 (0.070)
Ikaros				
Placebo	0.820	0.784 (0.030)	0.795 (0.033)	0.786 (0.033)
Iberdomide 0.15 mg QD	0.786	0.973 (0.042)	0.916 (0.045)	0.901 (0.042)
Iberdomide 0.3 mg QD	0.798	1.000 (0.032)	1.069 (0.036)	1.008 (0.034)
Iberdomide 0.45 mg QD	0.845	0.980 (0.030)	1.069 (0.033)	1.035 (0.033)

T cell exhaustion module				
Placebo	0.739	0.691 (0.015)	0.705 (0.015)	0.712 (0.017)
Iberdomide 0.15 mg QD	0.741	0.691 (0.020)	0.674 (0.021)	0.677 (0.021)
Iberdomide 0.3 mg QD	0.710	0.699 (0.015)	0.687 (0.016)	0.693 (0.018)
Iberdomide 0.45 mg QD	0.693	0.724 (0.015)	0.699 (0.016)	0.709 (0.017)
Ikaros Type 1 INF module				
Placebo	1.118	1.054 (0.055)	1.087 (0.058)	1.118 (0.067)
Iberdomide 0.15 mg QD	1.160	0.560 (0.076)	0.457 (0.079)	0.519 (0.082)
Iberdomide 0.3 mg QD	1.084	0.587 (0.056)	0.516 (0.061)	0.505 (0.069)
Iberdomide 0.45 mg QD	1.226	0.487 (0.055)	0.396 (0.057)	0.376 (0.060)
Aiolos				
Placebo	0.635	0.659 (0.028)	0.632 (0.029)	0.562 (0.029)
Iberdomide 0.15 mg QD	0.637	0.696 (0.039)	0.747 (0.039)	0.697 (0.040)
Iberdomide 0.3 mg QD	0.704	0.735 (0.029)	0.760 (0.031)	0.703 (0.033)
Iberdomide 0.45 mg QD	0.692	0.688 (0.028)	0.744 (0.030)	0.716 (0.030)

IFN, interferon; SE, standard error.

Supplementary Table 5. Numeric data for Figure 5: Clinical efficacy treatment comparison (Week 24 SRI-4 response rate, iberdomide 0.45 mg – placebo) within prespecified patient subsets defined by gene expression at baseline

Patient subset	Stratified Difference, %, SRI-4 at Week 24	95% CI
Overall	19.4	4.12, 33.42
Aiolos high	32.9	7.74, 52.90
Type 1 IFN high	26.8	7.49, 43.54
Ikaros IFN high	24.3	5.45, 40.76
T cell exhaustion low	21.9	4.30, 37.77
B cell high	20.4	0.74, 38.01
Ikaros low	21.5	–9.16, 48.45
B cell low	17.1	–7.45, 39.64
Ikaros high	16.1	–1.76, 32.65
Aiolos low	9.0	–10.11, 27.50
Ikaros IFN low	8.3	–17.65, 34.68
Type 1 IFN low	7.0	–18.01, 32.11
T cell exhaustion high	4.1	–24.69, 33.88

CI, confidence interval; IFN, interferon; SLE, systemic lupus erythematosus; SRI, SLE responder index.

Supplementary Figure 1. Population-predicted exposure by dose in A) all patients, B) patients by type I IFN signature at baseline and C) patients by Aiolos expression at baseline. The horizontal bold line in each boxplot represents the median value. The top and bottom edges of the box represent the 25th and 75th percentiles, respectively. Whiskers represent 1.5 × interquartile range. Dots are outliers. AUC_{ss}, area under concentration-time curve at steady state.

A.

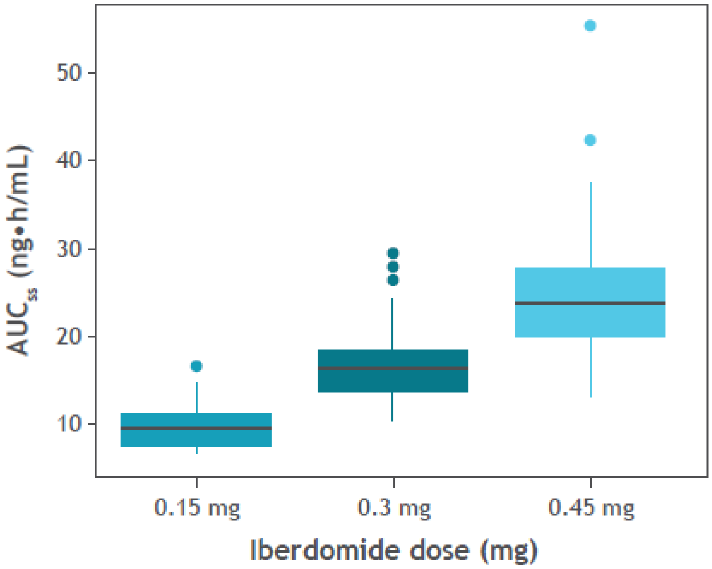

B.

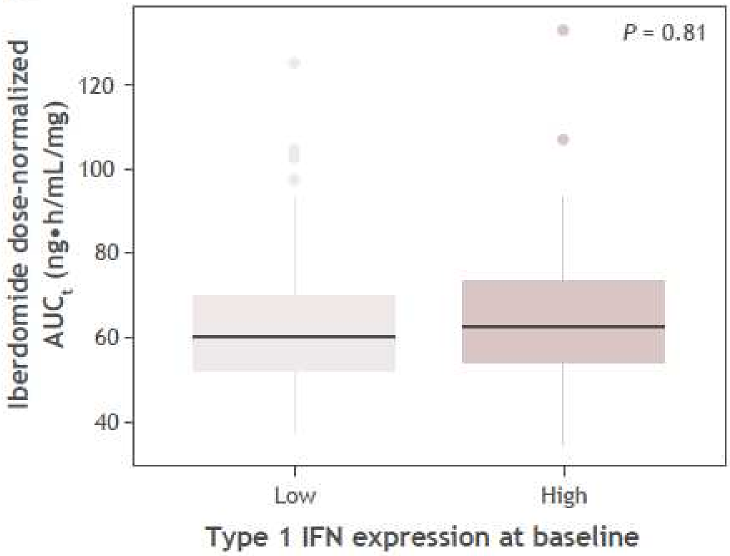

C.

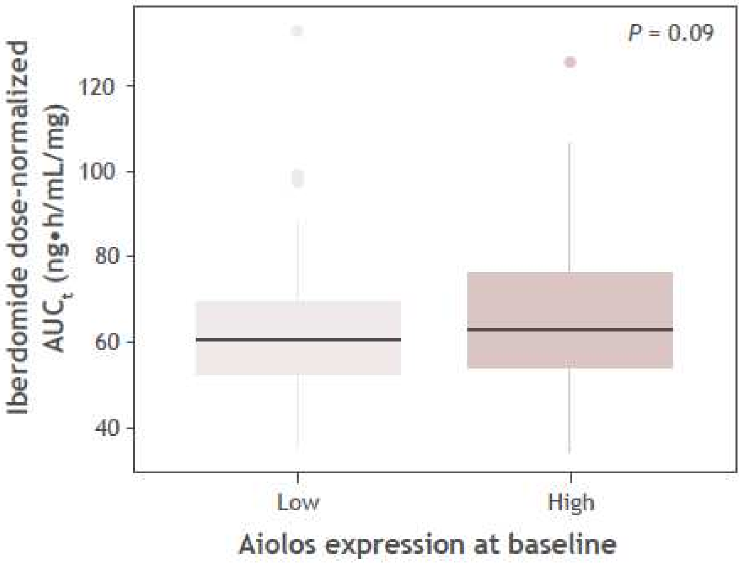

Lipsky PE, *et al. Ann Rheum Dis* 2022; 81:1136–1142. doi: 10.1136/annrheumdis-2022-222212

Supplementary Figure 2. Biomarker baseline and percent change from baseline values in IFN-Low (type I IFN genes ≤ −1.38) and IFN-High (type I IFN genes > −1.38) subsets for type I IFN signature score [*IFI27, IFI44, IFI44L, RSAD2*], CD303+ plasmacytoid dendritic cells, CD19+ B cells, interleukin-2, regulatory T cells, and double-stranded DNA antibodies (in patients with baseline dsDNA Abs ≥ 8 IU/mL). *p≤0.05, **p≤0.01,***p≤0.001.

From N Engl J Med, Merrill JT, Werth VP, Furie R, van Vollenhoven R, Dörner T, Petronijevic M, et al, Phase 2 trial of iberdomide in systemic lupus erythematosus, Volume No. 386, 1034-1045. Copyright © 2022 Massachusetts Medical Society. Reprinted with permission.

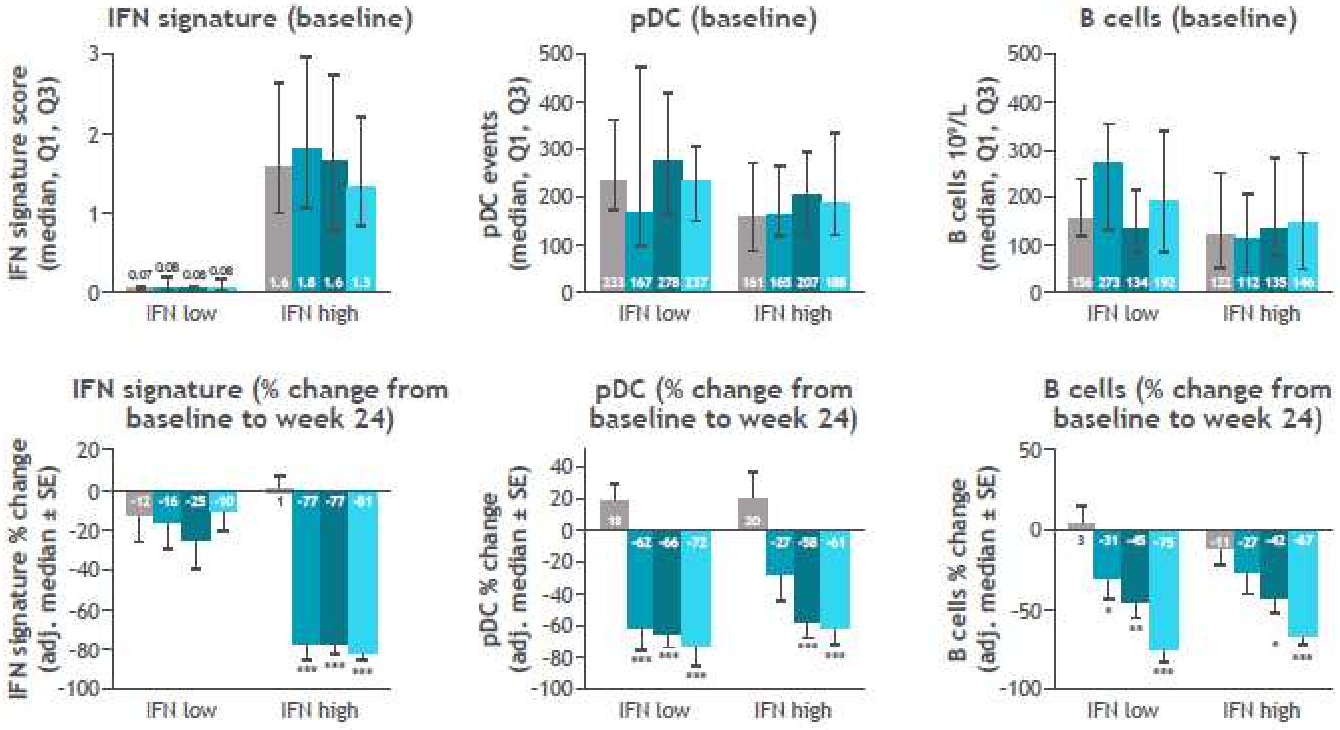

17

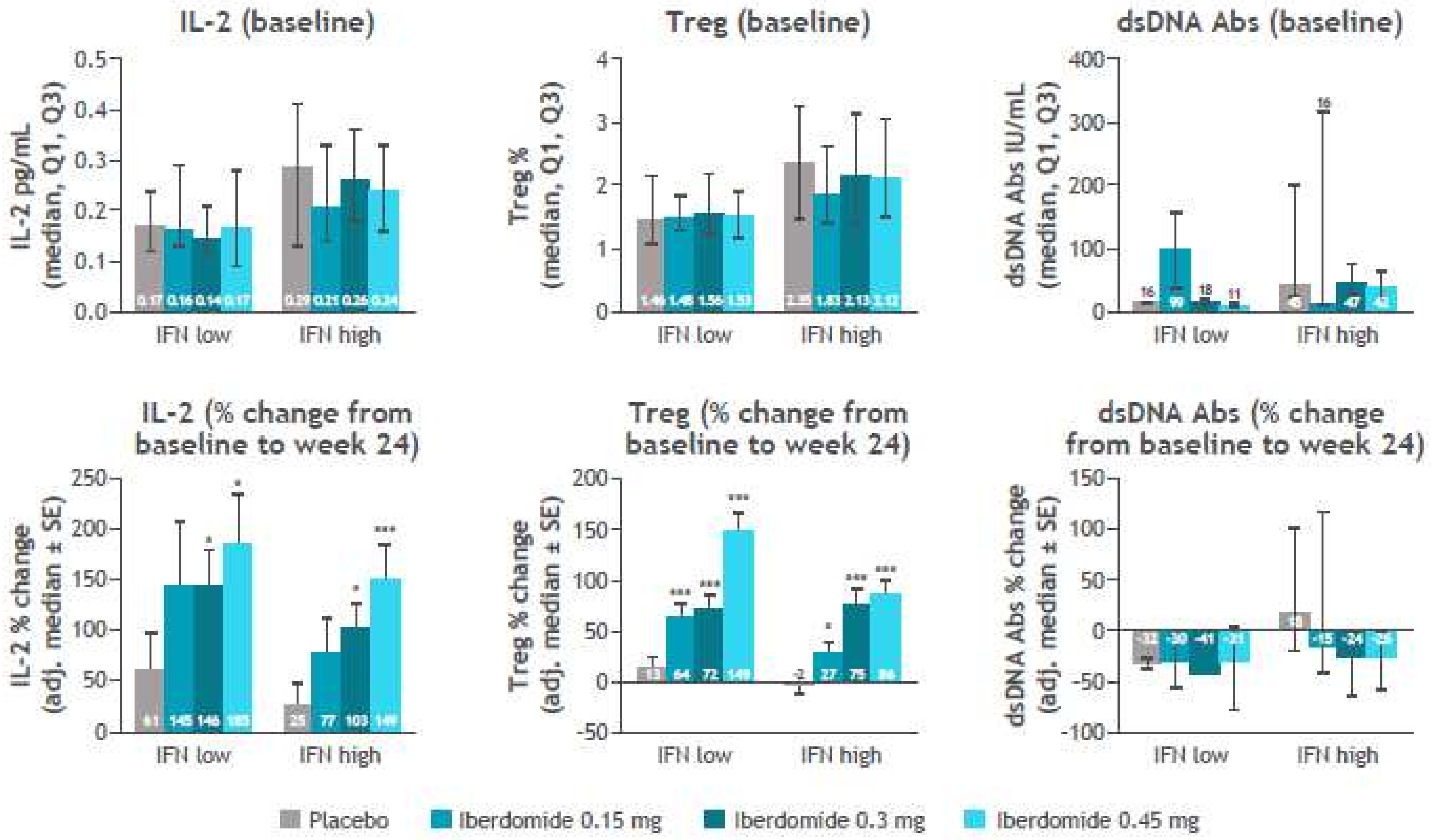

Lipsky PE, *et al. Ann Rheum Dis* 2022; 81:1136–1142. doi: 10.1136/annrheumdis-2022-222212

Supplementary Figure 3. Biomarker baseline and percent change from baseline values in Aiolos-Low (*IKZF3* ≤ −0.49) and Aiolos-High (*IKZF3* > −0.49) subsets: IFN signature score, CD303+ plasmacytoid dendritic cells, CD19+ B cells, interleukin-2, regulatory T cells, and double-stranded DNA antibodies (in patients with baseline dsDNA Abs ≥ 8 IU/mL). *p≤0.05, **p≤0.01,***p≤0.001.

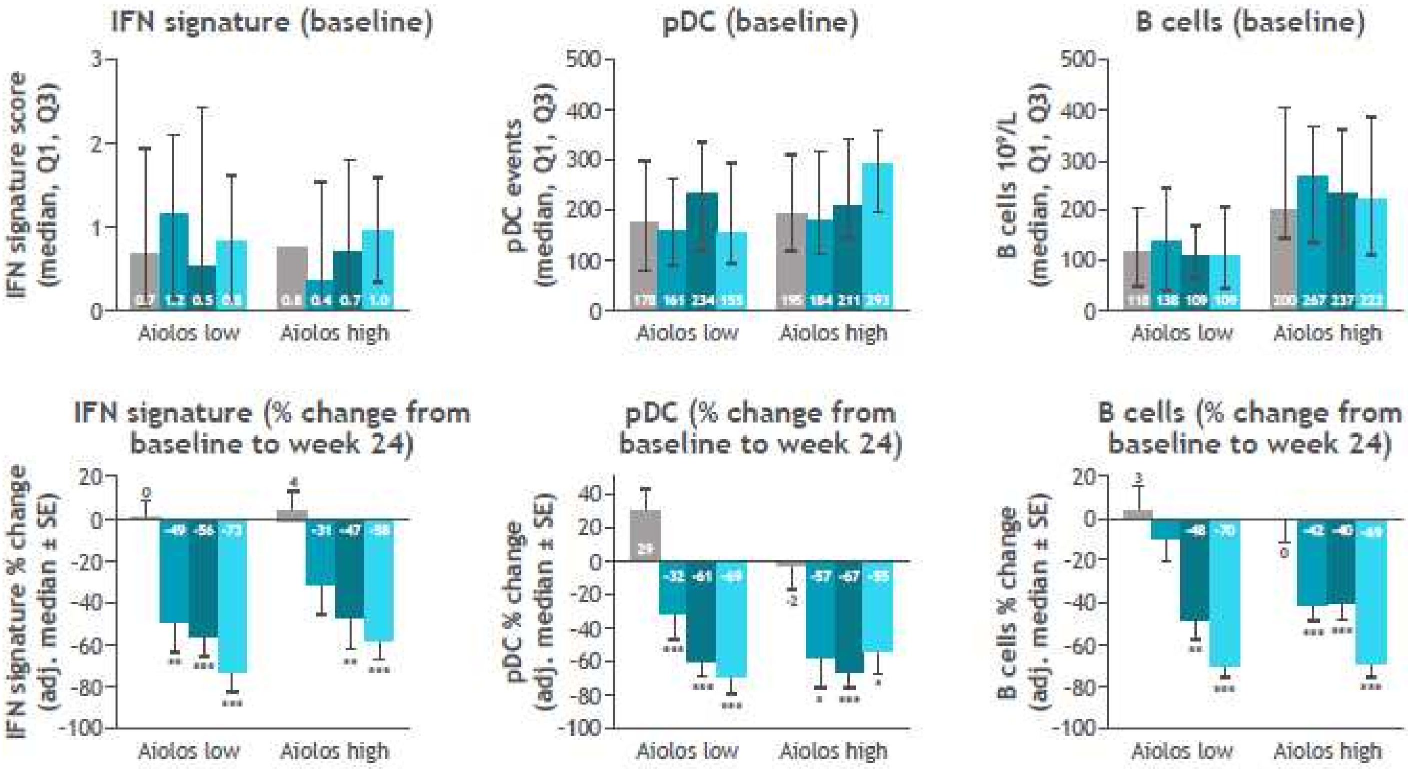

Lipsky PE, *et al. Ann Rheum Dis* 2022; 81:1136–1142. doi: 10.1136/annrheumdis-2022-222212

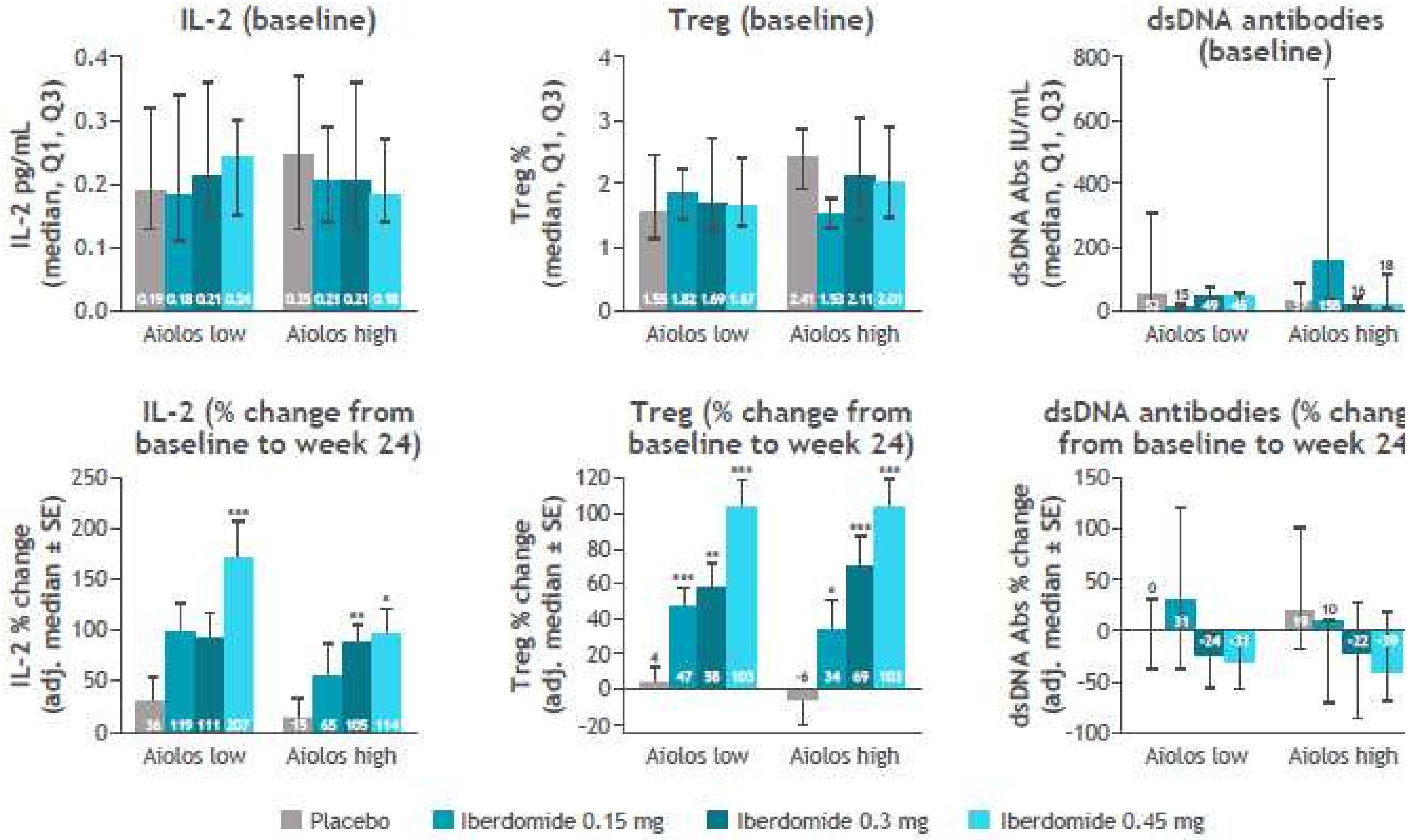

Lipsky PE, *et al. Ann Rheum Dis* 2022; 81:1136–1142. doi: 10.1136/annrheumdis-2022-222212
